# Multisystem Inflammatory Syndrome in Children Versus Neonates: A Case Series

**DOI:** 10.7759/cureus.76666

**Published:** 2024-12-31

**Authors:** Keshav Bhattar, Sudha S Anilkumar, Rashma Sadasivan, Ramesh Pandit, Nirali M Chaudhary, Trupti Pandit

**Affiliations:** 1 Internal Medicine, Dr. Sampurnanand Medical College, Jodhpur, IND; 2 Cardiology, Nemours Children's Health, Newark, USA; 3 Internal Medicine/Pediatrics, Western Michigan University, Kalamazoo, USA; 4 Medicine, Independent Researcher, Philadelphia, USA; 5 Hospital Medicine, University of Pennsylvania/Chester County Hospital, Philadelphia, USA; 6 Medicine, Hemchandracharya North Gujarat University, Ahmedabad, IND; 7 Pediatrics, Nemours Children's Health, Glen Mills, USA

**Keywords:** cardiac, children, covid-19, multisystem inflammatory syndrome, neonates

## Abstract

After several reports of a multisystem inflammatory disease in pediatric patients who had evidence of active or past infection with the novel coronavirus SARS-CoV-2, in May 2020, the Centers for Disease Control and Prevention (CDC) provided a case definition for this multisystem inflammatory syndrome in children (MIS-C). A similar presentation in neonates has been termed multisystem inflammatory syndrome in neonates (MIS-N). There is considerable overlap between clinical presentation for MIS-C and Kawasaki disease (KD) or atypical KD. Early diagnosis and treatment of these conditions are crucial to prevent serious complications. This case series describes three cases of MIS-C and MIS-N each and aims to provide evidence to advance the current understanding of these conditions.

## Introduction

In May 2020, the Centers for Disease Control and Prevention (CDC) provided a case definition for multisystem inflammatory syndrome in children (MIS-C) after several reports of a multisystem inflammatory disease presentation in pediatric patients with active or past infection with the novel coronavirus SARS-CoV-2 [1]. There is considerable overlap between clinical presentation for MIS-C and Kawasaki disease (KD) or atypical KD. KD and MIS-C are both rare conditions that cause inflammation throughout the body, and early diagnosis and treatment are crucial to prevent complications. These conditions have key differences including age groups involved, the association with infection, and evident long-term complications [2]. This case series describes three cases of MIS-C and multisystem inflammatory syndrome in neonates (MIS-N) each and aims to contribute to our understanding of these entities.

## Case presentation

We are presenting six cases, including three cases of newborns, one day old, two days old, and six days old, and three cases of pediatric age, one year old, two years old, and seven years old. Most of the patients are males except a seven-year-old female. 

Case 1 is a one-day-old male newborn born at 40 weeks of gestation by C-section due to fetal tachycardia. Mom was COVID-19 polymerase chain reaction (PCR) positive and acutely ill with COVID-19. The baby was less than the 10th percentile for weight and had tachypnea and tachycardia with desaturation on the first day. Labs showed elevated troponins. A fetal echocardiogram (echo) showed pericardial effusion on day 1 without a noticeable drop in cardiac function. The baby received IV immunoglobulin (IVIg) and steroids with continuous positive airway pressure (CPAP) for respiratory support. The echo finding of pericardial effusion resolved on day 4.

Case 2 is a two-day-old male newborn born by C-section due to cord prolapse. Mom was positive for COVID-19 PCR at the time of delivery. The baby developed tachypnea and tachycardia on the second day. There was granular opacity on the chest X-ray. Cardiac troponin (0.46) and pro-B-type natriuretic peptide (pro-BNP) were elevated. Echo showed moderate tricuspid regurgitation, coronary dilation, a reduced ejection fraction (EF) of 30-40%, and moderate right atrial and right ventricular dilation with supra-systemic right ventricular pressure (RVP) consistent with neonatal myocarditis as well as a possible thrombus in the right ventricle and pulmonary vein. The baby's creatinine was elevated to 2.2. The baby needed oxygen via high-flow nasal cannula and later CPAP due to respiratory distress. He also received two doses of IVIg, therapeutic Lovenox, captopril, milrinone, and aspirin.

Case 3 is a six-day-old male newborn born at 39 weeks via C-section. During a well-visit at six days, he was noted to have tachycardia up to 215 without fever or agitation. On investigation, he had elevated troponin (0.119) and BNP (5500). The mother was noted to have a positive COVID-19 reverse transcription polymerase chain reaction (RT-PCR) at the time of delivery. The baby's COVID-19 IgG was positive and the viral PCR was negative. Echo showed an EF of 43-54% on admission. An electrocardiogram (ECG) showed sinus tachycardia and a pause, with premature atrial contraction (PAC), which had a different P morphology. The baby was positive for COVID-19 IgG. He received IVIg, methylprednisolone, digoxin, enalapril, and acetaminophen. 

Case 4 is a one-year-old male patient who presented with intermittent fevers of 102°F for two weeks and abdominal pain and vomiting for two days. Both parents had COVID-19 about two weeks before the encounter. COVID-19 IgG was positive but viral PCR was negative on this patient. The patient's BMI was greater than the 99th percentile. Cardiac troponin and pro-BNP were elevated. Echo showed a low normal EF of 54% and atrioventricular (AV) valve regurgitation consistent with mild cardiac inflammation from MIS-C. D-dimer was elevated. The patient received two doses of IVIg and steroids. The MRI of the heart four months later was normal. 

Case 5 is a two-year-old male patient who presented with a fever (105°F) for six days associated with a progression of symptoms including rash, facial swelling, pink eye, sore throat, and leg pain. Troponins were 0.05 on admission and peaked at 0.16. He had BNP 341, C-reactive protein (CRP) 26, and anemia with H/H 8.6/26. Echo showed mild to moderate mitral regurgitation. COVID-19 IgG was positive. Viral PCR was negative. The patient received steroids, norepinephrine, Lovenox, and two doses of IVIg, along with oxygen 2 L/min via a nasal cannula. For suspected cellulitis and sepsis, he received ceftriaxone and vancomycin.

Case 6 is a seven-year-old female patient who presented with fever (Tmax 103°F) for four days, with vomiting ×3, decreased oral intake, and fatigue. She had a rash on the right side of her neck with pain and also a cough and congestion one week ago. The patient was negative for COVID-19 Epstein-Barr virus (EBV) IgG but her family members were reported to be COVID-19 positive one week prior. ECG was normal. Troponin on admission was 0.23 peaking at 0.69 in a few days. An echo was performed which showed mild tricuspid valve regurgitation and mildly depressed left ventricular (LV) systolic function. Abnormal lab results included sodium 129, CRP 80, and BNP >900. The repeat echo was within normal limits. The patient received clindamycin for suspected cellulitis. She received one dose of IVIg and steroids for MIS-C. 

All these patients' clinical presentations are summarized in Table 1 for comparison.

**Table 1:** Demographics and clinical presentation AMS: altered mental status; LN: lymph node; S/P ablation: status post ablation; SVT: supraventricular tachycardia; BMI: body mass index; CS: cesarean section; GBS: group B *Streptococcus*; SGA: small for gestational age; RT-PCR: reverse transcription polymerase chain reaction

Clinical features	Case 1	Case 2	Case 3	Case 4	Case 5	Case 6
Age	1 day	2 days	6 days	1 year	2 years	7 years
Sex	Male	Male	Male	Male	Male	Female
Ethnicity	Puerto Rican	Hispanic/Latino	Black	Hispanic/Latino	Middle Eastern	Hispanic/Latino
BMI/weight percentile	<10%	Weight 3.3 kg; at 19%	Weight 3.1 kg; at 32%	BMI 15.7; greater than the 99th percentile	BMI 13; 76th percentile	BMI 15; 54th percentile
Comorbidities	Low birth weight	None	None	Obesity	None	None
Perinatal history	Born at 40 weeks by CS d/t fetal tachycardia. Apgar 7, 8. Weight 3.1 kg. Mom is acutely ill with COVID-19+, GBS+, SGA baby	Born at 41 weeks by CS d/t prolapsed cord. Apgar 8, 8	Born at 38 weeks and 6 days by CS d/t breech. Good Apgar score	-	-	-
COVID-19 vaccination	None	None	None	None	None	None
Flu vaccination	None	None	None	None	None	None
Presenting symptoms						
Fever	-	-	-	+	+	+
Tachycardia	+	+	+	-	+	+
Tachypnea	+	+	-	-	-	-
Rash	-	-	-	-	+ (generalized)	+ (neck)
Pink eye	-	-	-	-	Bilateral	-
Headache/AMS	-	-	-	-	-	-
Cracked lips/red tongue	-	-	-	-	-	-
LN enlargement	-	-	-	-	-	Cervical
Conjunctivitis	-	-	-	-	+	-
Edema	-	-	-	-	+ (facial)	-
Nausea/vomiting	-	-	-	+	-	+
Abdominal pain	-	-	-	+	-	-
Duration of symptoms	1 day	1 day	Unknown	2 days	6 days	4 days
Past/family history	Mother COVID-19 RT-PCR+ at the time of delivery	Mother COVID-19 RT-PCR+ at the time of delivery	Mother COVID-19 IgG+; born at 39 weeks via CS	-	-	S/P ablation at birth due to SVT
Allergies	-	-	-	Animal dander, dust mite extract, lactose, shrimp	-	-

All these patients' laboratory test results and diagnostic test findings are presented in Table 2.

**Table 2:** Laboratory and diagnostic findings of the patients Neg: negative; RT-PCR: reverse transcription polymerase chain reaction; ECG: electrocardiogram; pro-BNP: pro-B-type natriuretic peptide; TLC: total leukocyte count; AST/ALT: aspartate and alanine aminotransferase; AR: aortic regurgitation; BMI: body mass index; CRP: C-reactive protein; EF: ejection fraction; ESR: erythrocyte sedimentation rate; HFNC: high-flow nasal cannula; IVIg: intravenous immunoglobulin; LA and RA: left and right atrium; LDH: lactate dehydrogenase; LVH and RVH: left and right ventricular hypertrophy; PAC: premature atrial contractions; PFO: patent foramen ovale; PR: pulmonary regurgitation; PDA: patent ductus arteriosus; WNL: within normal limits; USG: ultrasound; pro-TNF: pro-tumor necrosis factor; HHV: human herpesvirus; RVP: right ventricular pressure; MIS-C: multisystem inflammatory syndrome in children; HFrEF: heart failure with reduced ejection fraction; AB: antibodies; A: at admission; L: lowest; P: peak; ANA/RF: antinuclear antibody/rheumatoid factor; TR: tricuspid regurgitation; MR: mitral regurgitation; RPR: rapid plasma reagin; INR: international normalized ratio

Diagnostic findings	Case 1	Case 2	Case 3	Case 4	Case 5	Case 6
COVID-19 AB	NA	Neg	IgG+	IgG+	IgG+	Neg
COVID-19 RT-PCR	Neg (mother +)	All viral PCR: Neg	All viral PCR: Neg	NA	All viral PCR: Neg	All viral PCR: Neg
COVID-19 vaccination	None	None	None	None	None	None
Flu vaccination	None	None	None	None	None	None
ECG	LVH and repolarization abnormality, possible biventricular hypertrophy, prolonged QT interval 486 ms. Recent ECG Holter monitoring normal	-	Sinus tachycardia, few PAC	Possible RVH but no conduction, rhythm, or ST-segment change	Sinus tachycardia	Sinus tachycardia
Troponins (admission)	134	0.26	0.119	WNL	0.05	0.23
Troponins (peak)	569	0.45	0.119	0.07	0.16	0.69
Pro-BNP (peak)	992	86000	9300	39800	417	986
Hemoglobin	15.5	11.3	A 14; L 10.3	A 9; L 7.6	A 9.9; L 8.1	A and L 11
TLC	19.2	18.9	13	11.9	10.2	2.3
Lymphocyte %	NA	A and L 35	A 67; L 47	A and L 34.4	A and L 30	27
Platelets	346	146	121	149	63	297
Creatinine	0.3	2.2	0.3	0.3	0.2	0.5
Sodium	137	139	-	-	-	-
AST/ALT	Not done	105/26	33/13	61/33	42/18	58/36
LDH	Not done	645	Not done	Not done	686	A 161; P 865
Albumin	NA	3.1	3.2	2.5	2.3	3.3
ESR	NA	6	5	Not done	A 96; P 145	Not done
CRP	<0.3	Not done	After steroid treatment <0.5	A and P 9	A and P 26	A and P 8.9
Procalcitonin	NA	0.2	0.05	2.5	6.3	Not done
Ferritin	Not done	193	Not done	A and P 159	A 421; P 450	A 283; P 258
D-dimer	2000	0.23	Not done	A and P 2954	A and P 6504	A 3.7; P 1946
Fibrinogen	NA	A 155; P 244	Not done	A and P 548	A and P 470	608
Lactic acid	NA	Not done	1.2	1.5	A 0.8; P 2	Not done
IL-6	NA	Not done	Not done	Not done	<2	19
ANA/RF	Not done	WNL 0.2	Not done	Not done	Not done	Not done
Other lab work	-	Antithrombin III level, 72%. Plasminogen activity. Plasma, 67 mg/dL. Protein S level, normal. Factor V level, normal. Tested negative for the group B streptococcal infection, HIV, RPR, and hepatitis B virus. Blood group was O+, with Coombs Neg. INR level was 1.2	-	Lipase 1436; no pancreatitis on USG	IL-2=6283; IL-10=6	IL-2=3895; IL-10=15.7; pro-TNF=5.7; HHV <500 copies done due to cold sores during presentation
Echo	Small pericardial effusion and PFO on day 4. Normal LV and RV function. PFO small	Day 2: moderate TR and trivial AR, moderate RA and RV dilation with supra-systemic RVP, PFO (bidirectional), no PDA, moderately depressed systolic function EF 30-40%, no pericardial effusion. Day 5: thrombus RV and pulmonary vein, suspected thrombosis at LA appendages with normal LV and RV function and started on Lovenox for thrombus	WNL. EF 43-54% on admission	Low normal EF of 54% and AV valve regurgitation consistent with mild cardiac inflammation from MIS-C. Repeat: WNL	Mild to moderate MR but otherwise normal structure and function; no coronary artery dilation; confirmed MIS-C diagnosis. Echo prior to discharge showed stable mitral valve regurgitation. EF 75% and BNP 31	EF normal. No ectasia
MRI of the heart	Not done	Normal day 13	Not done	Normal 4 months later	Normal 7 months later	Not done
Primary diagnosis	Acute myocarditis	Acute myocarditis; RV and PV thrombosis; coronary dilation	Post-infectious myocarditis	MIS-C associated with COVID-19; HFrEF	MIS-C associated with COVID-19	MIS-C associated with COVID-19

## Discussion

The case definition for this MIS-C as described by the CDC is as follows: age <21 years presenting with fever (>38°C), laboratory evidence of inflammation (one or more of the following: an elevated CRP, erythrocyte sedimentation rate (ESR), fibrinogen, procalcitonin, D-dimer, ferritin, lactate dehydrogenase (LDH), or IL-6, elevated neutrophils, reduced lymphocytes, and low albumin), and evidence of clinically severe illness requiring hospitalization, with multisystem (>2) organ involvement and no alternative plausible diagnoses, and positive for current or recent SARS-CoV-2 infection by RT-PCR, serology, or antigen test, or COVID-19 exposure within four weeks before the onset of symptoms [1].

Such an inflammatory syndrome, when it occurs in newborns, has been called MIS-N. While there are no official diagnostic criteria, Pawar and colleagues have described provisional inclusion criteria [3]. In a case series of 20 neonates, they reported that 90% had cardiac involvement with prolonged QTc, AV blocks, cardiogenic shock, or coronary dilatation, 40% had respiratory failure, 10% had a fever, and 30% had feeding intolerance. In contrast, less than 5% had renal failure.

Notably, none of our patients with MIS-C had neurologic symptoms, which is in contrast to the previous series, which reported 4/6 patients with headache, altered mental status, irritability, or nuchal rigidity [4], which further denotes the current limited understanding of the incidence of neurological symptoms in MIS-C patients. The MIS-N cases in our report did not include fever, skin, or eye involvement. The MIS-N cases mostly involved cardiac systems with the elevation of troponin and other cardiac markers. None of our patients tested positive for SARS-CoV-2 by RT-PCR. However, 2/3 of the children and 1/2 of the neonates (results were not available for neonate 1) had COVID-19-specific IgG antibodies.

There is considerable overlap between MIS-C and KD. However, there are many differentiating features [2]. Classic features of KD, like polymorphous skin rash, bilateral bulbar conjunctival injection, oral mucous membrane changes, peripheral extremity changes, and cervical lymphadenopathy, if present at all, were preceded by fever and/or gastrointestinal (GI) symptoms in our patients. Extremity edema was not reported in our MIS-C patients. Two out of three of our patients with MIS-C had mild thrombocytopenia.

KD, particularly if untreated, is notorious for causing coronary artery aneurysms secondary to subacute to chronic necrotizing arteritis with a significant neutrophilic response [2]. Myocarditis and pericarditis can also occur [5]. Children with MIS-C can also have severe cardiac dysfunction at presentation, but that seems to be more commonly due to myocyte dysfunction (3/3 of children had elevated pro-BNP, and 2/3 of children had elevated cardiac enzymes) [6]. MIS-C can cause coronary aneurysms as well, but only 1/3 of our patients above age one year had such changes on echo. The key differences are detailed in Table 3.

**Table 3:** Comparison between KD, MIS-C, and MIS-N KD: Kawasaki disease; MIS-C: multisystem inflammatory syndrome in children; MIS-N: multisystem inflammatory syndrome in neonates; pro-BNP: pro-B-type natriuretic peptide

Clinical features	KD [6]	MIS-C [7]	MIS-N [2]
Cardiovascular	Coronary artery aneurysms. Myocarditis, pericarditis (rare)	Ventricular dysfunction with elevated pro-BNP and cardiac enzymes. Coronary artery aneurysms	Acute or post-infectious myocarditis
Pulmonary	Rare	Respiratory insufficiency, pleural effusions, and pulmonary infiltrates responsive to supplemental oxygen	Respiratory insufficiency
Gastrointestinal	Nausea, vomiting, abdominal pain, often presenting before the onset of classic symptoms	Similar to KD but more prominent	No gastrointestinal involvement in our patients
Hematological	Hypercoagulable state. Thrombocytosis	More hypercoagulability; high-dose aspirin with warfarin/enoxaparin indicated prophylactically; thrombocytopenia	D-dimer elevated in one case
Mucocutaneous/dermatological	Maculopapular rash, mucocutaneous ulceration including cracked red lips, strawberry red tongue, bilateral non-exudative conjunctivitis	Significant overlap (may meet the criteria for KD or atypical KD)	No skin involvement in our patients
Lymphatic	Cervical lymphadenopathy that is greater than 1.5 cm	Significant overlap (may meet the criteria for KD or atypical KD)	None of our patients
Renal	Rare	Common	Creatinine elevated in one newborn
Neurological	Sensorineural hearing loss, facial nerve palsy, severe irritability, and aseptic meningitis	Headache, altered mentation, rarely progressing to overt encephalopathy	No neuro involvement in our patients
Musculoskeletal	Arthritis and serositis may be present	Arthritis and serositis may be present	No joint involvement in our patients

Previous case series report that 8/8 and 4/6 of the children with MIS-C developed severe enteropathy [4,8]. Our series also depicts that 2/3 of children presented with GI symptoms like abdominal pain, nausea, and vomiting in the context of MIS-C.

Current treatment recommendations for MIS-C cases include IVIg, IV steroids, and additional measures based on individual patient scenarios [9]. Among our patients, 2/3 of children with MIS-C received IVIg and methylprednisolone, with excellent response, marked by the resolution of fever and improving cardiac function on subsequent follow-up.

The limitations of our study include a smaller sample size, which limits the generalization of findings to a broader population of children and neonates with MIS-C and MIS-N. Lack of statistical analysis, due to the study's descriptive nature, limits the ability to predict risk factors and outcomes. This article aims to report additional MIS-N cases, which should help define the new disease and its presentation better.

## Conclusions

We hope that with this case series, we further the knowledge of MIS-N. The CDC recommends that healthcare providers who have cared for or are caring for patients younger than 21 years of age meeting MIS-C criteria should report suspected cases to the respective health departments. We recommend pediatric providers to report suspected MIS-C or MIS-N cases, including patients meeting the criteria of KD, and especially MIS-N so the medical community can further the understanding of this emerging syndrome.
